# A non-invasive method for scoliosis assessment—A new mathematical concept using polar angle

**DOI:** 10.1371/journal.pone.0275395

**Published:** 2022-09-30

**Authors:** Susmita Roy, Alexander T.D. Grünwald, Renée Lampe

**Affiliations:** 1 School of Medicine, Klinikum Rechts der Isar, Orthopaedic Department, Research Unit of the Buhl-Strohmaier Foundation for Cerebral Palsy and Paediatric Neuroorthopaedics, Technical University of Munich, Munich, Germany; 2 Markus Würth Professorship, Technical University of Munich, Munich, Germany; Ningbo University, CHINA

## Abstract

Scoliosis is one of the most common pediatric spinal diseases that leads to a three-dimensional deformity of the spine and has a high risk of progression during growth. Regular clinical monitoring and follow-up X-rays are needed to providing proper treatment at that time. Repetitive X-rays can results in an increased risk of radiation related health problems. We present a non-invasive, ionizing radiation-free method for assessing scoliosis and its progression from the 3D images of the body torso, captured by a body scanner. A new concept is introduced based on a mathematical method in polar coordinate system to quantify and characterize the deformities in the torso from 2D transverse cross-sections of the 3D torso images at example cases for a healthy individual and for two patients with scoliosis. To capture quantitatively the characteristics of scoliosis, and to verify them at the example cases two asymmetry parameters and a linear fitting parameter are calculated: a) back side area asymmetry, b) left right area asymmetry, and c) coefficient of determination (*R*^2^). Within the analyzed patients, both the area asymmetries are maximum at the apex of scoliosis, and increase with the severity of scoliosis. *R*^2^ values are smaller in the case of patients compared with the healthy. Furthermore, the parameters show a trend when compared with the Cobb angle from the X-ray and the findings match with clinical examination. Therefore, the quantities are able to capture, certain characteristics associated with scoliosis. These quantities can be compared as a measure of deformities of torso, during the follow-up examinations in the future, without ionizing radiations.

## Introduction

A three-dimensional (3D) deformity of the spine of at least 10° lateral curvature in the coronal plane in combination with vertebral rotation around the vertical axis is defined as scoliosis [1]. The gold standard assessment for quantifying scoliosis is the Cobb angle, which is defined as the angle between the upper and lower end plates of the most laterally tilted vertebrae around the apex of the spinal curvature in a coronal view appeared in an X-ray image [2].

Children and adolescents with neurogenic and idiopathic scoliosis undergo regular radiological examinations to check the development of their spinal deformity during growth. As a result of serial X-ray examinations, young patients can accumulate significant amount of ionizing radiation over time. This radiation leads to the formation of oxygen free radicals, which can damage DNA and consequently lead to mutation. Therefore, the long term side effects associated with ionizing radiation can lead to an increase in breast, thyroid, and leukemia cancer risks [3, 4], especially in children who has a long life span, and who have rapidly dividing cells that may be more susceptible to developing DNA damage [5]. Various studies have shown that the incidence of cancer is higher in children with adolescent scoliosis who are often exposed to X-rays due to scoliosis, compared to healthy children of the same age [5, 6].

Magnetic resonance imaging (MRI) technology is widely used in medical diagnosis as a radiation free technique. MRI uses strong magnetic fields or magnetic field gradients and radio waves to produce two-dimensional (2D) cross-sectional images of the organs inside the body and provides anatomical information, therefore, the technique is radiation free. But, MRI can not be used solely for the assessment of scoliosis. Because the gold standard measurement of scoliosis, i.e. the Cobb angle, is measured in the frontal and sagittal planes from the X-ray images, can not be measured from MRI in a very straight forward way due to the differences in methods [7]. While an X-ray shows an overlay of the internal body structures, MRI images show a series of cross-sections. A coronary MRI image does not show the entire course of the spine due to its natural curvature. Another difference is that X-rays are usually taken in standing position, while MRI is done in lying position that can influence the degree of curvature of the spine. Additionally, MRI scanning procedure requires a long operating time that can be stressful for patients.

Therefore, there is a further need for assistive medical observation techniques which can reduce the number of ionizing X-ray examinations in scoliosis assessment. In the search for a radiation-free alternative, methods have emerged that assess scoliosis and its degree of curvature based on changes in the general body shape and outer body contour line. Generally, the lateral translations and rotations of vertebrae due to scoliosis yield outer torso deformations. Thoracic scoliosis normally becomes visible in a rib hump with posterior elevation on the convex side and a flattening on the concave side, due to the rotations of the vertebral bodies and the resulting distortion of attached ribs. In the case of lumbar scoliosis, waist asymmetry and a lumbar bulge are the most prominent signs. Therefore, it would be helpful to have characterization standards that can capture the shape of the deformed torso, e.g. quantifying the changes in rib hump prominence during the follow-up examinations of scoliosis, as an alternative to X-rays.

Surface topography is one of the non-invasive methods to investigate the shape of the back torso surface, where a deformed torso shape usually correlates with scoliosis [8–11]. However, the studies reported the results with various levels of success [12]. In some other external non-invasive radiation-free techniques, the positions of the spinous processes were determined from reflective markers attached to the skin over the processus spinous by different motion analysis systems such as Vicon, ZEBRIS ultrasound based system [13–15]. Their regression analysis and correlation coefficient results indicated that the method produced reliable results in sagittal and frontal curvatures, and could be used for follow-ups to control the effect of various therapies. However, the methods cannot replace the biplanar X-ray in the sagittal and frontal planes and does not provide much information about the rotation of vertebral bodies.

Recently, computer vision technology, which develops computer theories and methods from image information, has also been actively involved in the research area for the development of scoliosis assessment methods through visual information of human external anatomy. Using this idea, a 3D non-contact surface scanning device, hereafter mentioned as body scanner, has been developed with a camera system [16], especially for patients with cerebral palsy (CP) who suffer very often from scoliosis. The advantages of the clinical use of that body scanner [16], include its straightforward, easy operating system which is relatively fast. Additionally, the scanner is mobile. The fast scanning procedure is an advantage in comfort, especially for patients with disabilities like CP, because they have to hold their position and posture only for a short period of time (∼ 15 *sec*). The most important point is that the process involves no radiation exposure to the person being scanned. The body scanner provides a 3D image of the torso and 2D cross sections can be extracted from that along the vertical body axis.

The current study is a step towards developing an analysis method in the use of that body scanner for the clinical assessment of scoliosis in follow-up examinations. The concept of a new analysis method is presented here, with the objective of capturing and characterizing asymmetries, present in the 2D cross sections of the torso due to scoliosis. At the example of a couple of show cases, it is shown that the shape asymmetries in the 2D cross sections, extracted from the 3D scan image, are reflected in a change in values of some parameters presented here. These parameters can be compared as a measure of asymmetries of torso images taken at different times by the scanner during the follow-up examinations.

The basic idea behind the method presented here is based on performing the computation in polar coordinates and on the linear fitting of extracted body scanner data. The analysis procedure can quantify the asymmetry changes in each and every transverse cross sections along the vertical body axis, therefore, it is independent of growth and can be applied in particular also to adolescents. The advantage of this technique is in the quantitative parameterization of various transverse contour shapes in terms of angular coordinates, providing accurate and reliable results for scoliosis assessment.

## Materials and methods

### Experimental method and data

A concept of an automated analysis method is presented here at the example cases of 2D transverse cuts from four scan images of three different participants. The scan images of the participants were captured with a non-commercial, 3D body scanner [16]. The body scanner system has been previously designed and developed at the Chair for Computer Vision & Artificial Intelligence in the Faculty of Informatics at the Technical University of Munich. The main components of the 3D body scanner system were an ASUS Xtion Pro Live 3D depth camera system mounted on a swivel arm and corresponding software packages. The camera system consisted of a RGB color sensor and a combination of an infrared laser projector and infrared camera to measure the depth profile. During scanning, the camera system once circumnavigated the person, standing at the centre of a circle with 1.4 m diameter in the transverse plane. At the same time color images and depth profiles with a resolution of 640 x 480 pixels were captured in real-time with a frequency of 30 Hz [16]. The image data were then automatically (post-) processed by software packages, specifically developed for this end. As a result, the body scanner system provided a conformal 3D surface image of the person. The entire process of scanning and image reconstruction took about 15 seconds and was thus fairly quick and comfortable for the patient. Moreover, it was non-invasive and free from ionizing radiation. During scanning, participants were asked to stand still and upright with their arms slightly abducted. Scanning thus can be, in principle, repeated in the same way an indefinite number of times, and images and results derived from that are thus reproducible. Differences in participants’ height could be accommodated by adjusting the camera level accordingly. The analysis method presented hereinafter then detected torso asymmetries at the 2D transverse cross sections, resulting from scoliosis.

**Fig 1:**
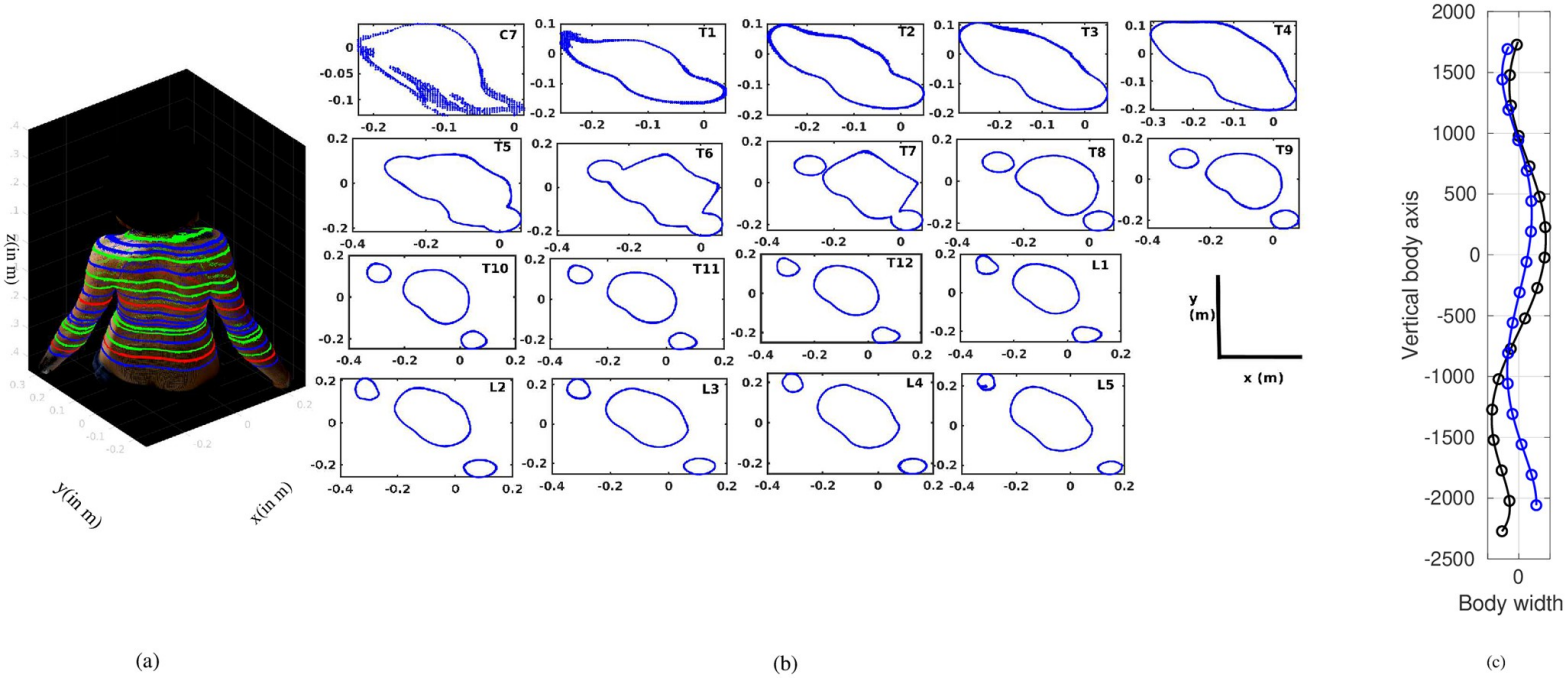
Scan image. (a) 3D scan image of a patient captured by a body scanner and the transverse body contours marked at different vertebral levels along the vertical body axis. (b) Extracted transverse body contours at vertebral levels from C7 to L5. C7 is the seventh cervical vertebra, T1–T12 indicates twelve thoracic vertebrae, L1—L5 are lumbar vertebrae. (c) Vertebral courses extracted from X-ray images: black line at the time of first visit, blue line at the time of one of the follow-up examinations.

Table 1 shows details about the participants’, analyzed here, in addition to the scoliosis assessment results from the patients evaluated by a senior pediatric orthopedic specialist from their X-ray images. One scan image was from a healthy person and another three were from two youth females with scoliosis (P1 and P2). As a standard diagnosis process, X-ray images were taken during the patient’s first visit, and Cobb angles of 30° (for P1) and 38° and 33° (for P2) were measured. Additionally, scan images were taken from both of them (P1F (Fig 1(a)) and P2). In the case of P1, after a brace treatment and scoliosis specific physiotherapy exercises, further, a body scan (P1S) and X-ray were done at the time of one of the follow-up examinations and the Cobb angle was found to be reduced to 20°. Extracted courses of the vertebral columns from the two X-ray images of P1 after [16, 17] are shown in Fig 1(c). In the case of P2, only one scan image was analyzed.

**Table 1:** Participant’s age, gender, height, weight and classification of their scoliosis evaluated from X-ray images.

Participant	Gender	Age year	Height in cm	Weight in kg	Scoliosis range	Apex position	Cobb angle in °
Healthy	Male	28	195	95	-	-	-
P1 (P1F) (1^*st*^time scan)	Female	14	170	52	thoracic	T8	30
P1 (P1S) (2^*nd*^time scan)	Female	16	182	60	thoracic	T8	20
P2	Female	17	168	58	combined	T9/L2	38/33

P1 and P2 indicate two patients. P1F: 1^*st*^ time scan of the patient P1, P1S: 2^*nd*^ time scan of the patient P1. T8 and T9 indicate the eighth and ninth thoracic vertebrae respectively. L2 indicates the second lumbar vertebra.

In principle, there were no exclusion criteria for scanning a participant with this body scanner. Even for the patients with CP, who often suffer from neurogenic scoliosis, with reduced ability to hold their position, the scanning procedure was fast enough (∼15 sec.) to capture clear images from them. Participation in this study was voluntary and written consents were obtained from participants, or their legal protectors, before the scanning session. Data were pseudo—anonymous and all procedures were approved by the ethics committee of the Faculty of Medicine of the Technical University of Munich before starting the study (Ref 569/16 S).

### Analysis of torso image

The present method analyzed independently 2D transverse cross-sections of the body contour at each vertebral level, from C7 to L5 along the vertical body axis.

The scan images analyzed here were inspected by a senior orthopedic specialist and C7 (extreme top), T8 (middle), and L5 (extreme bottom) vertebral levels were identified on that, at the beginning of the analysis process. 2D transverse sectional planes cutting the torso at these vertebral levels, as well as at the other estimated vertebral levels in between were extracted then similar to the slicing approach employed by Roy et al. [18], in which transverse segmental torso slices were extracted from computed tomography (CT) images. Each of these 2D cross-sections, referred to here as slice, contained the contour line of the torso at that vertebral level and was analyzed separately from all other slices. An example of a scanned image together with marked vertebral levels (left panel) and slices of the torso extracted for analysis at each vertebral level from top to bottom (middle panel) are shown in Fig 1.

Upon extracting slices from the 3D scan images, computational algorithms were applied to find characteristic and specific points of those slices (e.g. centroid, spinous process, left-right sides), which were then further used to quantify the shape asymmetries of those slices. The algorithm performed the following mathematical steps (described below) and deducted automatically the quantities, throughout the vertical body axis. The computation time required for this analysis, for all vertebral levels, was around 1 min. Next we start by defining and describing the mathematical steps, before defining the asymmetry parameters. The whole analysis method was developed using Matlab2020a (The MathWorks, Inc., Natick, MA, USA).

### Analysis of the slices

Extracted slices at all the vertebral levels from C7 to L5 (Fig 1(b)) were automatically and separately analyzed following the method described below.

#### Coordinate system and definition of certain points

**Fig 2:**
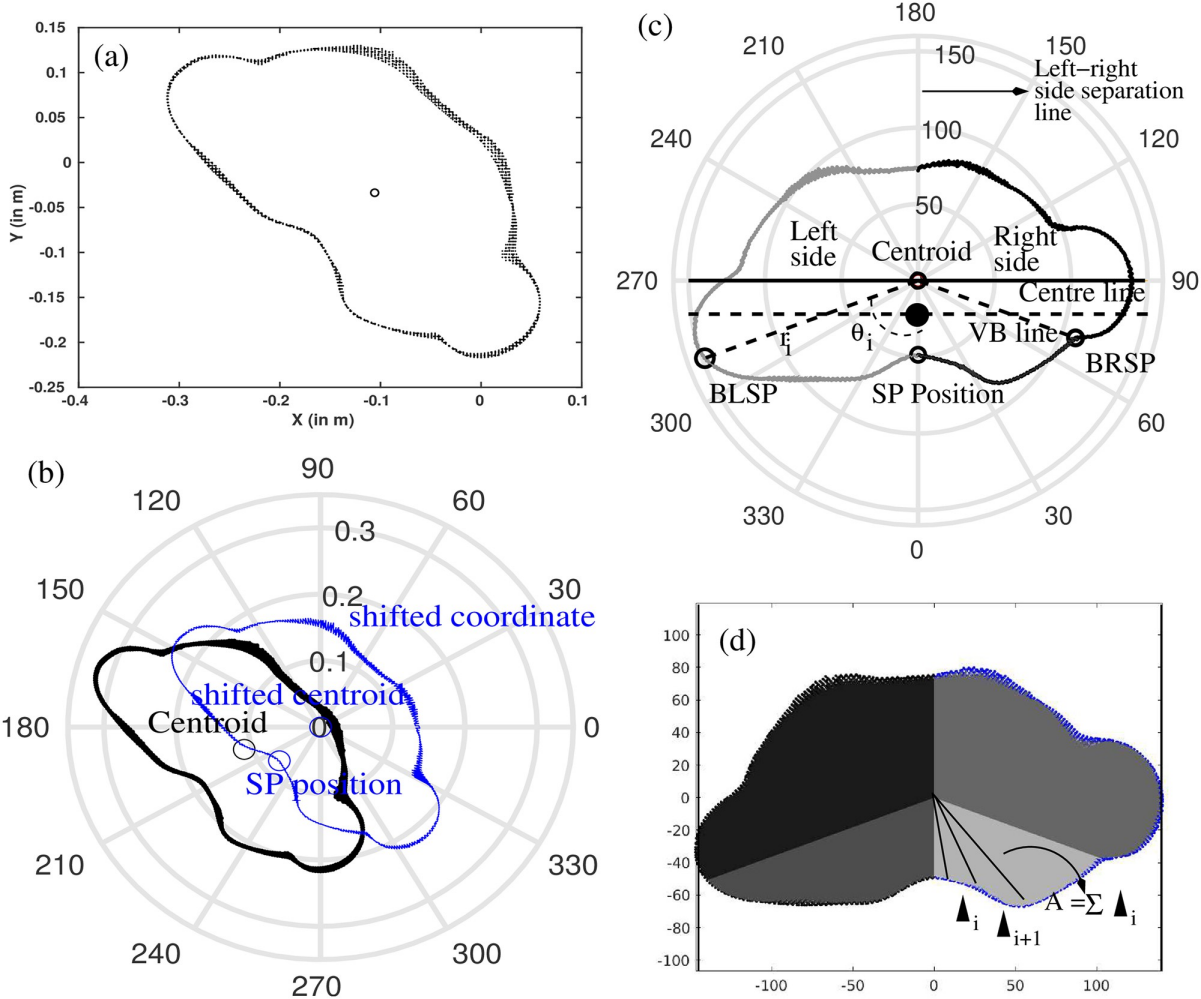
An example slice with specific points. An example transverse cross section of the torso. (a) The original transverse cross-section in Cartesian-coordinates extracted from the scan image. (b) The contour converted into polar coordinate system. The contour with black points represents the original one, the contour with blue points represents the translated one where the centroid coincides with the origin of the polar coordinate system. The centroid of either contour are marked with ‘o’ symbol. (c) The rotated and rearranged slice is represented in terms of relative radius. Also represented are specific points (Centroid, SP position, BRSP, BLSP) and regions (Right side, Left side) that are required for the calculation of the symmetry parameters (see main text for more details). (d) The considered areas in the calculation.

- Coordinate transformation: The coordinate system of the transverse cross sections was converted from Cartesian (*x*_*i*_, *y*_*i*_) to polar coordinates (*r*_*i*_, *θ*_*i*_) (Fig 2(a) and 2(b)), so that the shape of the slices could be analyzed as a function of the angular distribution of contour radii *r* = *r*(*θ*) [19], where *θ* ∈ [0° − 360°) was the polar angle defined with respect to the polar coordinate system shown in Fig 2(b).
- Centroid extraction: The centroid of a slice was defined as the arithmetic mean of all the points of that contour belonging to that particular cross-section (black contour in Fig 2(b)). Then, the coordinates of the cross-sections were translated in such a way, that the centroid coincides with the origin of the polar coordinate system (blue contour line in Fig 2(b)), and shifted new coordinate points were sorted in terms of their polar angle.
- Spinous process position extraction: The location of the spinous process was assumed to coincide with the characteristic dip on the lower section of the contour line (marked ‘SP Position’ in Fig 2(b)). This position was found by the minimum radius of the contour for each slice.
- Rearranging the slice: Next, each slice was rotated in a two-step process: First, the maximum diameter of the slice passing through the centroid was considered as a central line and made parallel to the horizontal axis of the coordinate system. Second, the SP position was fine-tuned so that the centroid and the SP position were aligned on the same vertical line. Finally, the right and the left sides of the slice were assigned with respect to the SP position, which means, points with angular coordinates from 0°/360° (i.e. SP position) to 180° refer to the right side and points in between 180° and 360° refer to the left side, respectively. Parts not belonging to the torso contour, e.g. the part of hands extracted along with the torso contour at the lower vertebral levels were removed manually from the calculation (from T8 level in Fig 1(b)). Fig 2(c) shows an example slice along with the afore defined specific positions.
- Vertebral body line (VB line): To estimate the approximate position of the centre of vertebral body inside a slice, the spinous process length was considered after [20], but the anatomical shapes of the vertebral bodies were neglected. The position was shown in Fig 2(c) with the filled black circle point. A line parallel to the center line and crossing through the approximated center of the vertebral body was marked as VB line.
- Backside left and right points: Two points at an equal polar angle distance from the spinous process were located for detailed analysis of the backside. The two points were marked in Fig 2(c) as BLSP (back left SP) and BRSP (back right SP) respectively.

#### Definition of parameters

- Relative radius: In order to analyze the shape of the slice each contour radius *r*_*i*_ = *r*(*θ*_*i*_), (*i* = 1, 2, …*n*) was normalized with respect to the mean contour radius:
  $$\begin{array}{ccc} r_{m}=\frac{1}{n}\sum_{i=1}^{n}r_{i} & = & \frac{1}{n}\sum_{i=1}^{n}r\left(\theta_{i}\right) \end{array}$$ (1)
  where, n was number of calculated radius *r*_*i*_, and polar angle, *θ*_*i*_, assumed equal distant values in steps of 1° in between −180° and + 180°; i.e. *θ*_*i*_ = 0°, ±1°, ±2°… ±180°. Instead of a function of individual contour radii *r*(*θ*_*i*_) the analysis was performed in terms of a non dimensional relative parameter
  $$\begin{array}{ccc} RR & = & \frac{r(\theta_{i})}{r_{m}}\times 100; \end{array}$$ (2)
  Since SP position coincided with 0° and 0 to 180° line divided the slice into left and right segments, symmetry analysis could be performed by analyzing the relative radii of left and right sides.
  $$\begin{array}{c} RR_{i}^{right}=\frac{r(\theta_{i}^{right})}{r_{m}}\times 100;\;\left(\theta_{i}^{right}=0,1,2\ldots 180^{{}^{\circ}}\right) \end{array}$$ (3)
  $$\begin{array}{c} RR_{i}^{left}=\frac{r(\theta_{i}^{left})}{r_{m}}\times 100;\;\left(\theta_{i}^{left}=0,-1,-2\ldots .-180^{{}^{\circ}}\right) \end{array}$$ (4)
- Coefficient of determination (*R*^2^): Theoretically, for a perfect geometrical symmetry, it followed:
  $$\begin{array}{c} RR_{i}^{right}=RR_{i}^{left}. \end{array}$$ (5)
  This means that if a linear function was created that satisfied the condition
  $$\begin{array}{c} RR_{i}^{right}=RR_{i}^{left}, \end{array}$$ (6)
  i.e. *y* = *x*, it represented a virtually, ideally symmetrical slice. In consequence, the closer the actual points $\left(RR_{i}^{right},RR_{i}^{left}\right)$ were to the symmetry line (*y* = *x*), the better was the symmetry of the contour. Mathematically, this criterion could be quantified using a coefficient of determination (*R*^2^), which was defined as:
  $$\begin{array}{c} R^{2}=1-\frac{\sum_{i=1}^{n}{\left(y_{i}-{\hat{y}}_{i}\right)}^{2}}{\sum_{i=1}^{n}{\left(y_{i}-\bar{y}\right)}^{2}}, \end{array}$$ (7)
  where *y*_*i*_ and $\hat{y}$ represent the actual (the values obtained from contour points) and fitted values of relative radii, respectively. $\bar{y}$ represents the mean of *y*_*i*_. *R*^2^ falls between 0 and 1. The higher the value of *R*^2^, the better the symmetry between the left and right sides.
- Left-right area asymmetry: The area of the regions spanned by the corresponding contour line and located to the left and right of the spinous process position (defined before as left and right side) were computed. In order to capture the exact shape of the areas to the left and right side of the separation line, the regions were split into small triangles (▴_*i*_). The centroid point and two further consecutive points on the contour line formed the vertices of a triangle and were used to compute the area of each triangle. Finally, the sum of all the triangle areas provided the area of the required portions, *A* (Fig 2(d)), so that
  $$\begin{array}{c} A=\sum {▴}_{i}. \end{array}$$ (8)
  The area asymmetry parameter was computed based on the areas to the left and the right side of the spinous process, *LA* and *RA* respectively, as follows:
  $$\begin{array}{c} abs\;(\frac{LA-RA}{LA+RA}) \end{array}$$ (9)
  Both of the parameters (*R*^2^ and left-right area asymmetry) were calculated considering the whole left and right side regions and for the segments SP to BRSP and SP to BLSP.
- Distance to VB line (*Dist*_*VB*_): Vertical distances on either side of the spinous process (from SP to BRSP and SP to BLSP) were computed from the vertebral body line (VB line) and defined as *Dist*_*VB*_.

## Results

**Fig 3:**
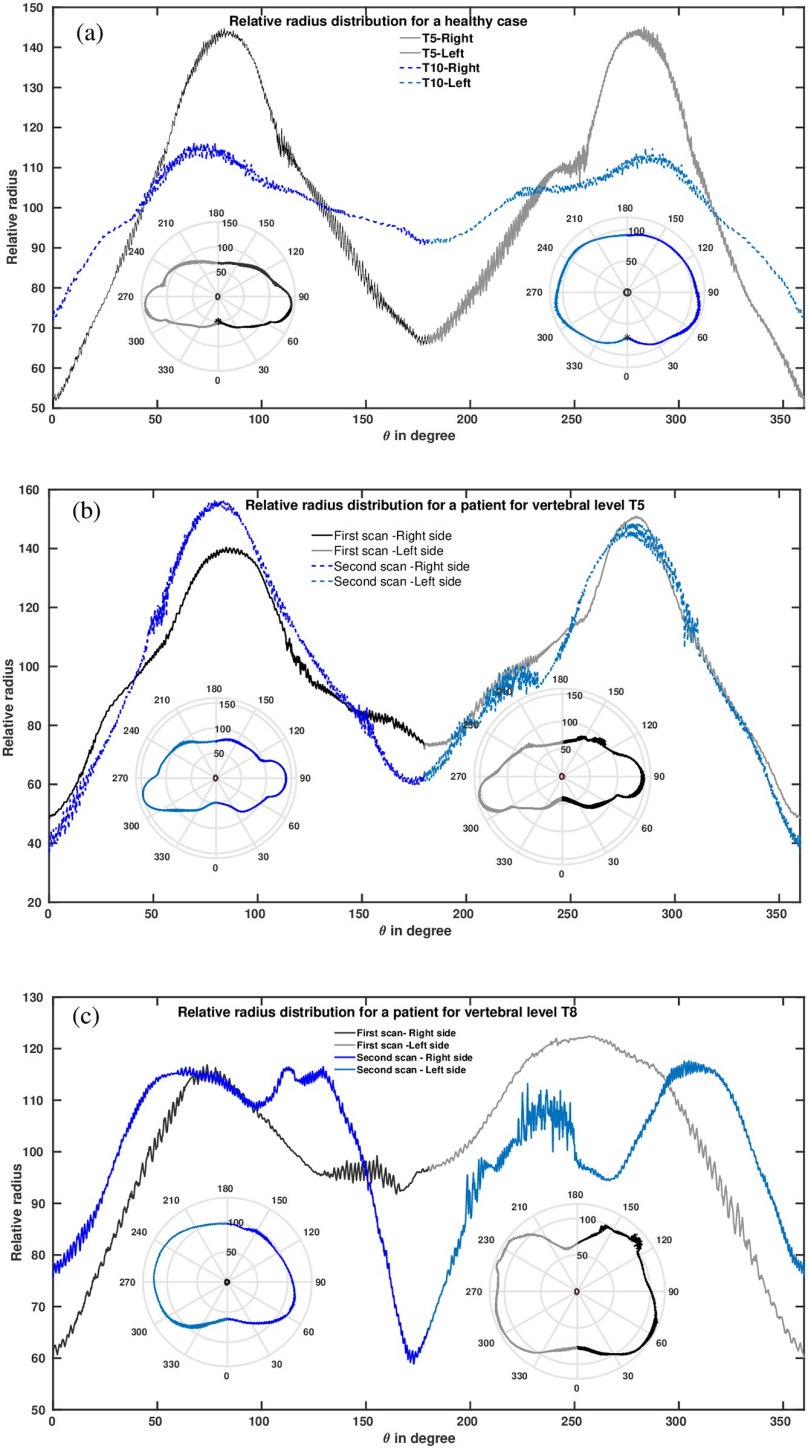
Angular distributions of example slices. (a) Examples of angular distribution from the transverse torso contours in polar and in linear coordinate, for the example cases extracted at two different vertebral levels in case of a healthy person. The black and gray lines are at the T5 level, the blue and light blue are from the same person at the T10 level. (b) Examples of angular distribution from the transverse torso contours in polar coordinates and also in linear coordinates, at the T5 vertical level in case of a person with scoliosis. The black and gray lines are from the person at the T5 level during her first scan. The blue and light blue from the same person but during a follow-up check. (c) The same as (b) but for the level of T8. The insets at all the subplots show the corresponding contours in polar coordinates.

Fig 3(a)–3(c) represent the normalized relative radii (evaluated following the Eq 2), of the contours as a function of angle, in linear and in polar coordinates, (a) for the case of a healthy at T5 and T10 level, and, (b) and (c) for the case of patient P1. Fig 3(b) and 3(c) overlaps the relative radii from P1F and P1S at T5 and T8 level, respectively, for P1. The working principle is illustrated for these example slices, but was independently applied also to all other vertebral levels. With this angular distributions the different shapes of slices were characterized quantitatively and tried to relate with scoliosis. Since the deformities at the back could be a sign of scoliosis, some results are also presented giving emphasis on both sides of the spinous process up to 70° angular separation positions (BRSP and BLSP in Fig 2(c)).

**Fig 4:**
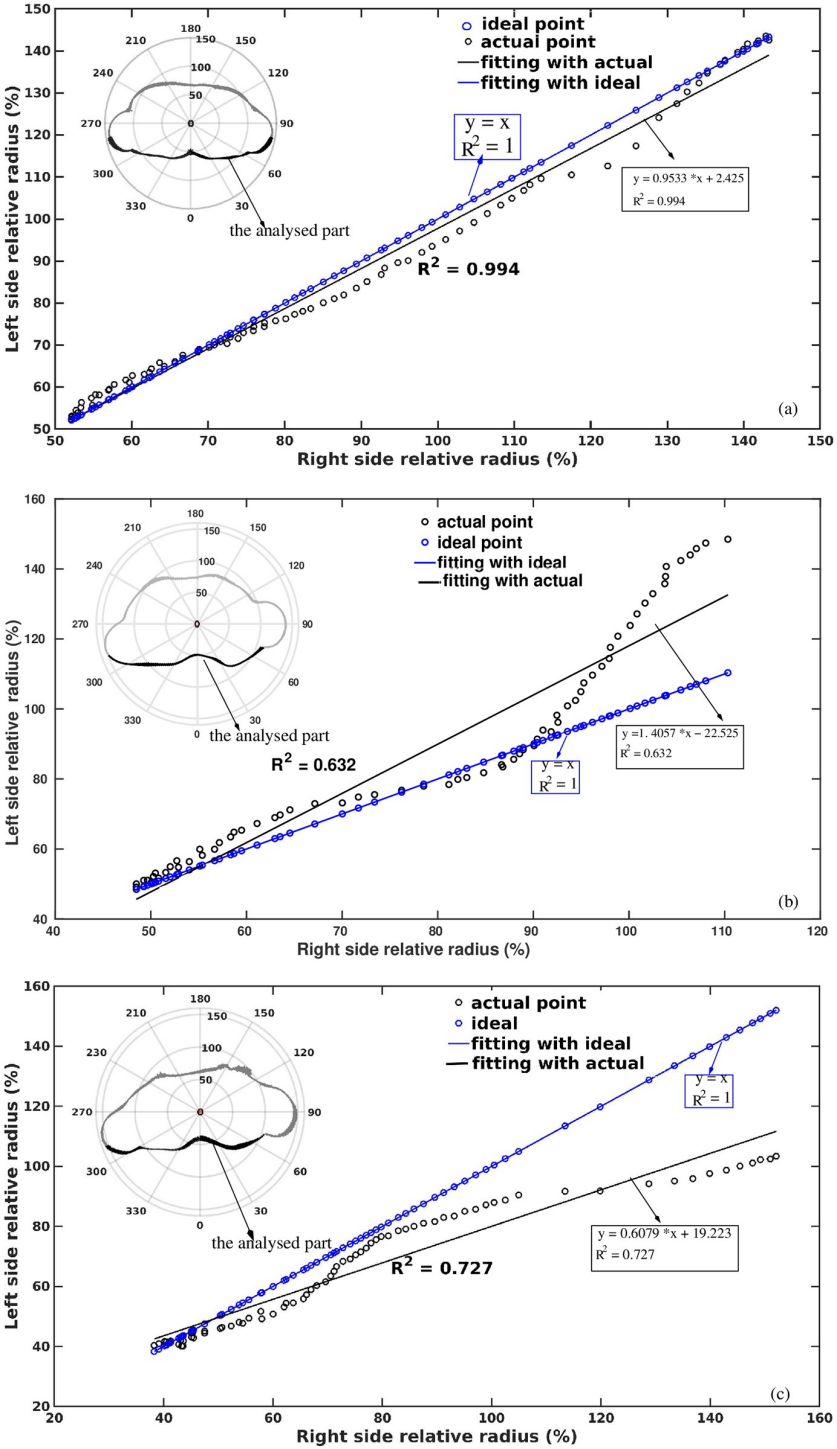
Correlation between two back sides. Correlation between the left and the right side of the normalized back side radii of the 2D transverse cross-section of the torso, (a) from healthy control, (b) and (c) from the patient at the time of her first scan and during a follow-up examination respectively. Black circles are the extracted original scattered points from the contour, blue circles indicate the ideal (symmetrical) shape. The linear trend of the transverse section has: a black line indicating the original shape, and a blue line indicating the ideal symmetric shape. The insets show the corresponding transverse cross-sections. Indicated are also the values of *R*^2^, that characterize the asymmetry of the backside torso contour shown here.

Fig 4 shows the correlation between the left and right back side normalized radii of the slices presented in Fig 3(a)–3(c). The considered angular ranges are highlighted in the insets of Fig 4. To create an ideally symmetric slice, one side data (let’s say left side) of the slice were mirrored and analyzed with respect to the actual (non-mirrored) left-side data. Therefore, in that case, all the contour points coincided with the theoretical line (Eq 6, blue lines in Fig 4) and yielded the value of *R*^2^ = 1.

Fig 4(a) represents the case for the healthy at the T5 level, where the comparison of both sides’ back contour points satisfies the linear equation,

$$\begin{array}{c} y=0.9533*x+2.425 \end{array}$$ (10)

with *R*^2^ = 0.9945. On the other hand, the linear fit through the normalized radii of the contour points at the T5 level shown in Fig 4(b) and 4(c) for the case of the patient (P1) for two different scans (P1F and P1S), respectively, follows the linear equations:

$$\begin{array}{c} y=1.4057*x-22.525 \end{array}$$ (11)

$$\begin{array}{c} y=0.6079*x+19.2239 \end{array}$$ (12)

with *R*^2^ = 0.632 and 0.727, respectively. In the case of the patient, a lower *R*^2^ value is a direct consequence of large data dispersion around the ideal line. Furthermore, the slope of 0.9533 (which is close to 1) in Eq 10, corresponds to the back side of a healthy person, which is symmetrical in shape. On the other hand, the slopes of 1.4057 and 0.6079 in Eqs 11 and 12 related to deformed back sides. Figures display also the virtually ideal distributions of the back sides (blue plots).

**Fig 5:**
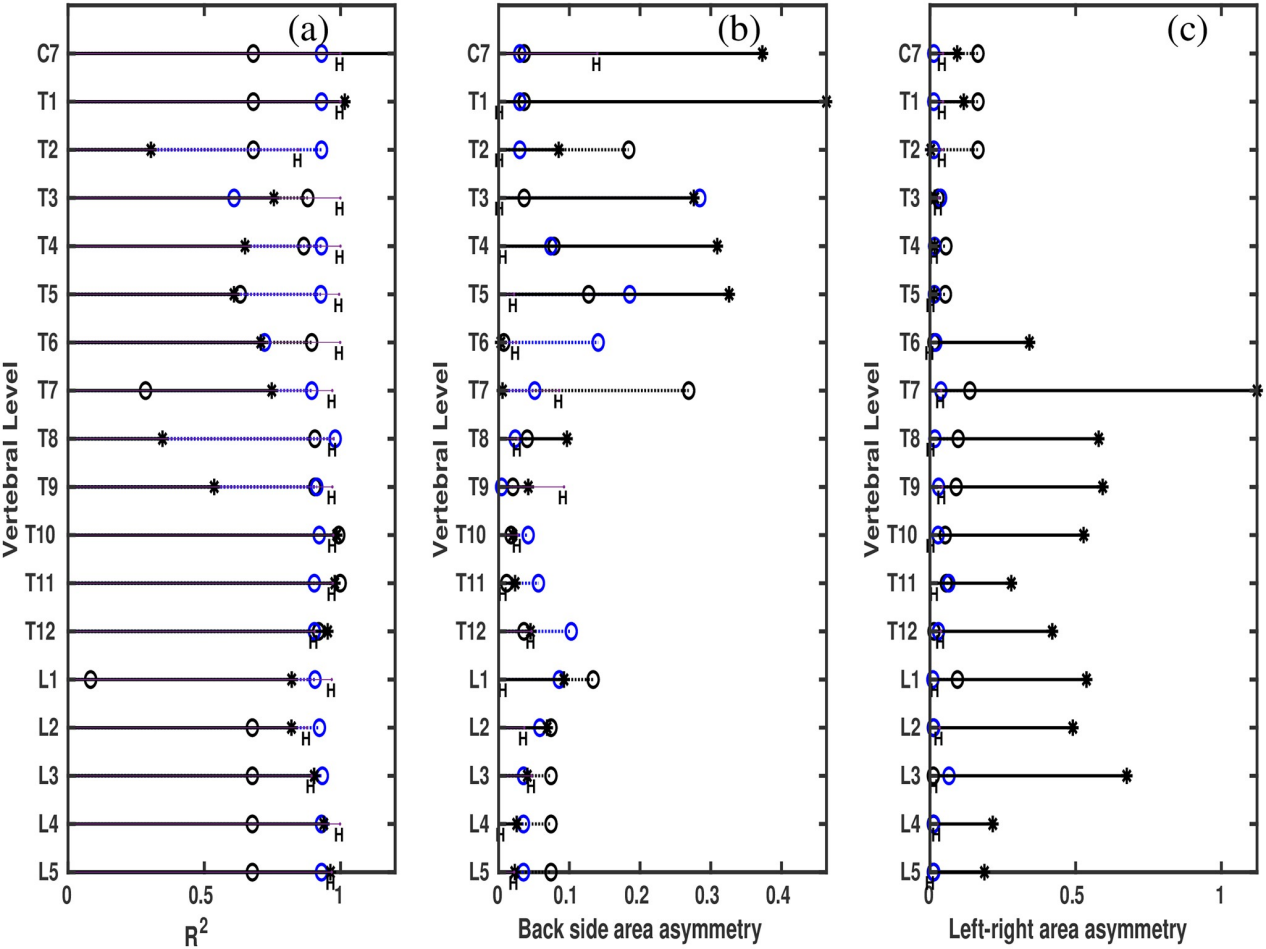
Asymmetry parameters along vertical body axis. Asymmetry parameters at different vertebral levels for the cases analyzed here. The letter ‘*H*’ represents the value for the healthy participant, ‘*o*’ for the case of P1 from her first scan, ‘o’ for the case of P1 at her second scan, ‘*’ for the case of P2. (a) Shows *R*^2^ values, (b) back side area asymmetry values, (c) left-right area asymmetry values.

Computed *R*^2^ values for different vertebral levels are plotted in Fig 5(a) for all the cases analyzed here. Different symbols represent individual data sets. The plot shows *R*^2^ values are near to one in the case of healthy (letter ‘H’) and generally, smaller in the case of patients. Smaller *R*^2^ indicates more asymmetries between the left and right sides of the slices. Fig 5(b) and 5(c) illustrate in turn the back side and total left-right area asymmetry, at different vertebral levels for the cases analyzed here. Represented are the individual area asymmetry values. The smaller the values of these two parameters, the more symmetric the slices are from the left and right sides. The plot shows, generally, for the case of the healthy these parameters are very small, compared to the cases of scoliosis. Especially, the total left-right area asymmetry (Fig 5(c)) shows larger values for more severe cases for the cases analyzed here.

In the case of P2, medical assessment as well as X-ray analysis revealed the regression of scoliosis. Especially the change in left-right area asymmetry values (Fig 5(c)) captured this assessment clearly.

Table 2 summarizes the parameters, indicating also the apex regions. The range of vertebral levels at the apex regions was selected according to the range covered by the lateral curvature of the spinal column. Table 2 shows the following distinct observations: 1) The asymmetry values are larger for the patients compared to the healthy control. 2) The asymmetry values are larger and *R*^2^ values are smaller in general in case of severe scoliosis. Further, smaller asymmetries on average and at the apex region and bigger *R*^2^ were expected in case of P1S.

**Table 2:** Mean of the evaluated asymmetry parameters for all the levels and at the apex.

Participants	Cobb angle in deg	Back side area asymmetry		Left-right area asymmetry		*R* ^2^	
		average of all levels	average at apex region	average of all levels	averageat apex region	average of all levels	averageat apex
Healthy	-	0.046	NA	0.0215	NA	0.9610	NA
P1 at the time of first scan (P1F)	30	0.0739	0.0876	0.0683	0.0737	0.7280	0.7692
P1 at the time of second scan (P1S)	20	0.0724	0.0745	0.0248	0.0245	0.8153	0.8928
P2	38	0.1288	0.0388	1.3833	2.3655	0.7066	0.5844
	33	0.1288	0.0672	1.3833	2.2701	0.7066	0.8123

**Fig 6:**
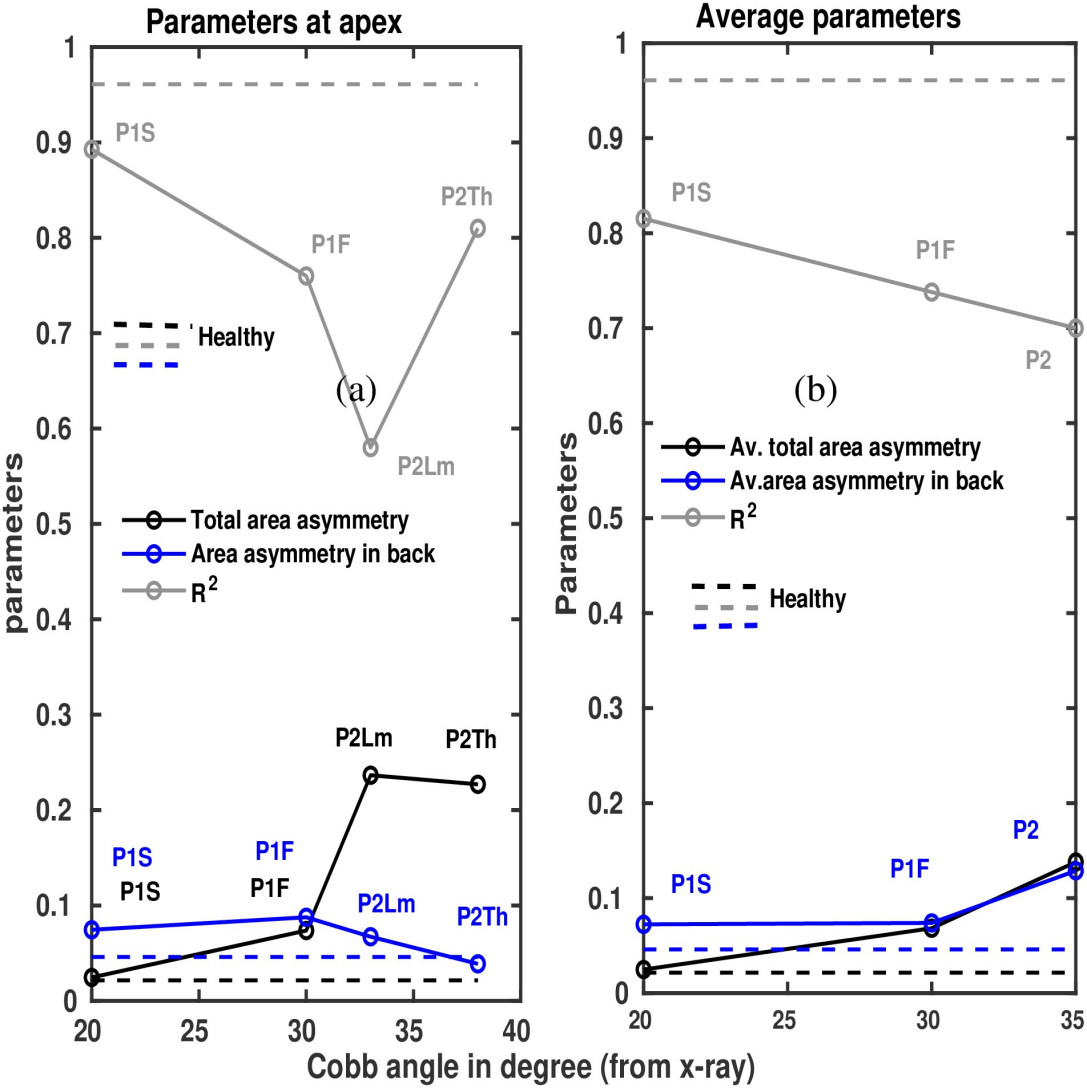
Asymmetry parameters vs Cobb angle. (a) Obtained asymmetry parameters are plotted against extracted Cobb angles for the cases analyzed here. Lines with symbols represent the values in patients. Dashed lines indicate the healthy case. Different colours indicates different parameters. P1F indicates the value of the parameter for P1 at first scan, P1S indicates the value of the parameter for P1 at second scan, P2Th indicates the same for P2 at thoracal region, P2Lm the same at lumber region. (a) Represents the values at apex, (b) average values from different vertebral levels.

Fig 6 illustrates the graphical view of Table 2 against Cobb angle. In general, the figure shows that *R*^2^ decreases with increasing Cobb angle and area asymmetry increases with increasing Cobb angle. The Figure also shows the healthy lines, which represent the values in case of the healthy for the corresponding parameters.

**Fig 7:**
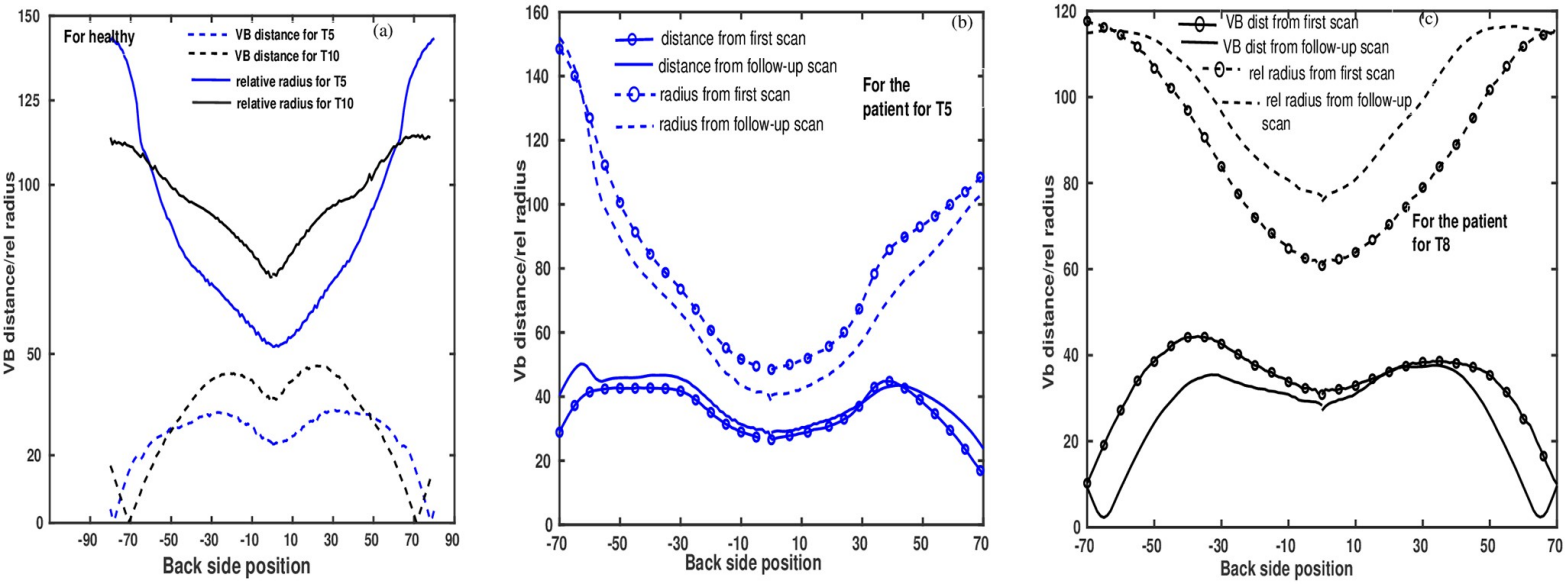
Back side asymmetries. Back side relative radius and distance from vertebral body line to contour points are presented, (a) from healthy control, (b) and (c) from the patient P1 at T5 level and T8 level, respectively.

Fig 7(a)–7(c) illustrate *Dist*_*VB*_ and back side radii (*Rad*_*back*_) for the healthy (Fig 7(a)) and for the patient P1 (Fig 7(b) and 7(c)). Represented are the individual distributions of *Dist*_*VB*_ and *Rad*_*back*_, on both sides of SP for back sides only for two different vertebral levels. The local minimum point of the distribution (0 position) represents the SP point. For the healthy case, the distribution is quite symmetric around the SP point. On the other hand, the distributions are clearly asymmetric for the case of the patient. This distribution also allows one to compare the change in characteristics of backside contours at different times.

## Discussion

The current work presents a new concept of a method developed to (1) analyze 2D transverse cross-sections extracted from a 3D scan image of a human torso (Fig 1(a)) and (2) quantify characteristics of scoliosis from the asymmetries of those extracted cross-sections. The purpose of this development is to combine the analysis method with the scanner system to use that as a technology, free of ionizing radiation, in the follow-up examination of scoliosis. Three quantities are calculated to capture the asymmetries of the 2D cross sections. The quantities are a) back side area asymmetry, b) left-right area asymmetry, and c) coefficient of determination (*R*^2^). Within the analyzed subjects, the results show that the quantities change their values with the state of scoliosis. Therefore, the present method might have the potential to reduce the number of X-rays during the monitoring of scoliosis. For initial diagnosis, as well as in the cases where surgical intervention is considered, however, the present method cannot replace the conventional and established imaging techniques (e.g. X-ray, MRI). Since detailed information of the spinal structure and the patient’s specific anatomy of the inner body is provided by these images and most of the treatment algorithms are based on them. Nevertheless, in contrast to the conventional methods, the present system may be used in the follow-up examinations of scoliosis, as an ionizing radiation free, fast, routine check-up imaging system in standing position.

Precise extraction of the vertebral levels (Fig 1) from the 3D scan image is an important step to locate the same vertebral level at the time of follow-ups. Detection of spinous process position by finding the dip in the back part of the contour (Fig 2) is also important because the further calculations are based on the position of the spinous process. In fact, correspondence of the characteristic dip on the contour line with the spinous process position and the capture of contour asymmetry with respect to the spinous process position was studied in a previous research with CT data [16, 18]. The results showed that the characteristic dip on the back surface of the human torso constitutes a good approach to assuming the tip of the spinous process position. This finding was also supported by Little et al. [21], where the dips on the back surface were marked with external markers, assuming that these are the anatomical landmarks of the spinous processes positions. Later on, the trajectories of the markers were compared with the positions of spinous process locations measured from MRI scans and obtained a good matching between the two positions. These studies support the development of the current method for the use of the body scanner. Furthermore, the advantage of the current method is that it does not require setting markers on the torso of the patient.

The method can analyze and quantify the entire as well as any part of the transverse torso contour shape (Fig 3) in detail by choosing the specialist’s ‘region of interest’, provided that, the physicians would have a graphical user interface (GUI) [22]. Additionally, like surface topography methods, the present method can quantify the rib prominence (Fig 7). By analyzing the shapes of the extracted transverse contours independently at different vertebral levels, the method is not affected by natural growth. Making a GUI of this method will be the next step to bringing the body scanner into clinical practice.

Within our analyzed samples both the area asymmetries and the *R*^2^ (Figs 5 and 6) show a trend with the severity of scoliosis and also with respect to the healthy person. In particular, larger values of area asymmetries, especially, total left-right area asymmetries are obtained at the apex regions and when the state of the scoliosis is more pronounced (see also Table 2). These are here the cases of patient P1 at her first time scan (P1F) and patient P2. The asymmetries in the contours of transverse sections of the torso were already associated with scoliosis and verified with CT [18]. Here, in the case of slices from scanner, the pattern of change in asymmetry quantities with the severity of scoliosis indicates that the quantities are able to capture from the transverse sections of the body torso to some extent, characteristics associated with scoliosis. Analysis of more scoliosis cases is necessary to efficiently characterize the different types and severeness of scoliosis and to bring the scanner into regular clinical practice.

Despite the gaining acceptance of modern low-dose biplanar radiography imaging systems, EOS [23, 24], still it is difficult to use them for children with disability due to its requirement to maintain a very stable position. Further, the EOS system is expensive, requires a large space and radiation shielding, thus its accessibility is not very high to everywhere. In addition to the two-dimensional information obtained from X-rays, a three dimensional understanding of spinal deformities is found to be useful and important by the orthopaedics in the treatment of scoliosis [25]. In this regard, various optical surface measurement systems, such as, Moiré topography, that uses interference patterns generated by a light source and a line grid on the back of the patient [26]; rasterstereography [9], that use stereophotogrammetric surface measurement of the back based on Moiré topography, are used [27–29]. Furthermore, 3D ultrasound imaging methods have also widely used for scoliosis assessment [30–33]. Moreover, several studies have evaluated the relationship between foot pressure distribution and the degree of scoliosis by analyzing gait and plantar structure of the foot [25, 34, 35]. All these methods without ionizing radiation, can either reconstruct the spinal curvature with reasonable accuracy or correlate different measuring constants with the degree of scoliosis. However, some of these systems use markers on the anatomical landmarks on the back surface, some of them often require a trained operator because of the rather complicated setup, but more or less can reconstruct the curvature of the spine with different processing times.

To predict the exact locations of the vertebral bodies inside the slices, similar like CT and MRI images, and to account for their rotational effects on the shapes of the slices, as well as to reconstruct the curvature of vertebral column further developments are required for the present study. This is expected to be accomplished with the integration and simulation of the rib-cage model [17, 22, 36]. In summary, the present method, based on mathematical calculations, has advantages in quantifying the changes in each and every transverse cross sections at different vertebral levels with time without markers, in a short time, in standing position and independently with patients’ natural growth. Therefore, in addition to clinical examination, it supports a decision against/for doing another X-ray at that moment, and thus has the potential to reduce exposure to ionizing radiation in follow-ups of scoliosis.

## Conclusion

A concept of scoliosis assessment method by analyzing 2D transverse cross sections of body contour from a 3D body scan image, in polar coordinate system, is presented in this paper. The analysis method first estimates the vertebral levels from the scan image and then extracts 2D transverse contours for each level. The transverse sections are then adjusted to facilitate further analysis. In the next steps, computations for accurate physical analysis of contours’ geometrical properties are computed. Finally, the method includes some quantitative asymmetry parameters to characterize scoliosis. Further, testing on a large number of data sets with diverse types of scoliosis is necessary to bring the method to the clinical application of scoliosis assessment.
